# Extended evaluation on the ES-D3 cell differentiation assay combined with the BeWo transport model, to predict relative developmental toxicity of triazole compounds

**DOI:** 10.1007/s00204-015-1541-6

**Published:** 2015-06-06

**Authors:** Hequn Li, Burkhard Flick, Ivonne M. C. M. Rietjens, Jochem Louisse, Steffen Schneider, Bennard van Ravenzwaay

**Affiliations:** Division of Toxicology, Wageningen University, Tuinlaan 5, 6700 HE Wageningen, The Netherlands; Experimental Toxicology and Ecology, BASF SE, Z 470, 67056 Ludwigshafen, Germany

**Keywords:** Developmental toxicity, Embryonic stem cell test, Placental transfer, BeWo cells, Alternatives to animal testing

## Abstract

The mouse embryonic stem D3 (ES-D3) cell differentiation assay is based on the morphometric measurement of cardiomyocyte differentiation and is a promising tool to detect developmental toxicity of compounds. The BeWo transport model, consisting of BeWo b30 cells grown on transwell inserts and mimicking the placental barrier, is useful to determine relative placental transport velocities of compounds. We have previously demonstrated the usefulness of the ES-D3 cell differentiation assay in combination with the in vitro BeWo transport model to predict the relative in vivo developmental toxicity potencies of a set of reference azole compounds. To further evaluate this combined in vitro toxicokinetic and toxicodynamic approach, we combined ES-D3 cell differentiation data of six novel triazoles with relative transport rates obtained from the BeWo model and compared the obtained ranking to the developmental toxicity ranking as derived from in vivo data. The data show that the combined in vitro approach provided a correct prediction for in vivo developmental toxicity, whereas the ES-D3 cell differentiation assay as stand-alone did not. In conclusion, we have validated the combined in vitro approach for developmental toxicity, which we have previously developed with a set of reference azoles, for a set of six novel triazoles. We suggest that this combined model, which takes both toxicodynamic and toxicokinetic aspects into account, should be further validated for other chemical classes of developmental toxicants.

## Introduction

The EU REACH legislation requires the safety assessment for new and existing chemicals. At present, regulatory safety assessment is predominantly performed using animal models, with large numbers of animals needed particularly for developmental toxicity testing. Therefore, REACH stimulates the use of animal-free approaches wherever possible (Hartung 2009; The European Parliament and the Council 2006). Alternative test methods for in vivo developmental toxicity testing, accepted for use in regulatory toxicity testing, are urgently needed (Höfer et al. 2004). The mouse embryonic stem D3 (ES-D3) cell differentiation assay is a valuable tool that can be used to predict in vivo developmental toxicity potency rankings within selected chemical classes for most of the chemicals investigated so far (De Jong et al. 2009; Louisse et al. 2011; Strikwold et al. 2012). However, as this assay does not take kinetic processes into account, it was suggested to combine the ES-D3 cell differentiation assay with data on kinetics, e.g., placental transfer of compounds, in order to better predict in vivo potency of the developmental toxicants (De Jong et al. 2009; Louisse et al. 2011; Strikwold et al. 2012). We have previously shown that an in vitro BeWo transport model is capable of predicting relative placental transfer rates of a set of nine model compounds with a good correlation with the relative transport rates observed in the ex vivo placental perfusion model (*R*^2^ = 0.95), indicating that the in vitro BeWo transport model is useful to obtain data with respect to placental transfer of compounds (Li et al. 2013). When combining the BeWo model with the ES-D3 cell differentiation assay, for a set of reference azoles, the coefficient of determination (*R*^2^) for correlation of relative in vitro potency with relative in vivo potency increased from 0.57 to 0.95 (Li et al. 2015), showing that the combined approach is able to better predict the in vivo developmental toxicity of azole antifungal compounds than the stand-alone ES-D3 cell differentiation assay.

Our first study included data on five reference antifungal compounds, with four of them being azoles. The azole family represents the largest family of antifungal compounds, which can be subdivided into the imidazole and triazole groups. We selected the class of azoles, because of their widespread use as antifungal agents in medicine and crop protection (Menegola et al. 2013) and because they are known to cause developmental toxicity, for which an extensive toxicity data base is available, required for evaluation of predictions made. The reference azoles used demonstrated a varying degree of developmental toxicity (Marotta and Tiboni 2010; Stinchcombe et al. 2013), allowing for a quantitative assessment of potency, which was the basis of the first validation of the combined ES-D3 and BeWo assay strategy.

The aim of the present study is to further validate the combined ES-D3 cell differentiation assay and BeWo transport model for predicting in vivo potency of developmental toxicants. To this end, six novel triazoles (Table 1) were investigated in the combined in vitro approach and the obtained predictions for these compounds were compared with the in vivo data that are presented in this paper for the first time, not taken from the literature.

**Table 1:** Chemical information of six triazoles tested in the present study

Code	Name	MW (g/mol)
0594	[5-(4-Chloro-2-fluoro-phenyl)-3-(2,4-difluoro-phenyl)-isoxazol-4-yl]-pyridin-3-yl-methanol	416.8
0595	[2,4-Bis-(2,4-difluoro-phenyl)-thiophen-3-yl]-pyridin-3-yl-methanol	415.4
0596	4-{2-[2-(4-Fluoro-phenyl)-2-hydroxy-1-methyl-3-[1,2,4]triazol-1-yl-propyl]-thiazol-4-yl}-benzonitrile	419.5
0599	4-[2-[2-(4-Fluorophenyl)-2-hydroxy-3-imidazol-1-yl-1-methyl-propyl]thiazol-4-yl]benzonitrile	418.5
0600	1-[[2-[2-Chloro-4-(4-chlorophenoxy)phenyl]-4,6-dimethyl-1,3-dioxan-2-yl]methyl]-1,2,4-triazole	434.3
0618	4-Methyl-1,3-dioxolan-2-yl-methyl-1,2,4-triazole derivate	440.0

## Materials and methods

### Chemicals

BASF triazoles 0594 ([5-(4-chloro-2-fluoro-phenyl)-3-(2,4-difluoro-phenyl)-isoxazol-4-yl]-pyridin-3-yl-methanol), 0595 ([2,4-bis-(2,4-difluoro-phenyl)-thiophen-3-yl]-pyridin-3-yl-methanol), 0596 (4-{2-[2-(4-fluoro-phenyl)-2-hydroxy-1-methyl-3-[1,2,4]triazol-1-yl-propyl]-thiazol-4-yl}-benzonitrile), 0599 (4-[2-[2-(4-fluorophenyl)-2-hydroxy-3-imidazol-1-yl-1-methyl-propyl]thiazol-4-yl]benzonitrile), 0600 (1-[[2-[2-chloro-4-(4-chlorophenoxy)phenyl]-4,6-dimethyl-1,3-dioxan-2-yl]methyl]-1,2,4-triazole) and 0618 (4-methyl-1,3-dioxolan-2-yl-methyl-1,2,4-triazole derivate) were kindly provided by BASF SE (Ludwigshafen, Germany). Dimethyl sulfoxide (DMSO) was purchased from Acros Organics (Geel, Belgium).

### BeWo transport experiments

To study placental transfer of test compounds, BeWo transport experiments were performed as described previously (Li et al. 2013). Briefly, BeWo b30 cells (passages 27–45) were cultured in DMEM (Zwijndrecht, The Netherlands), supplemented with 10 % (v/v) heat-inactivated FCS (HyClone-Perbio, Etten-Leur, The Netherlands), 10,000 U/ml penicillin, 10 mg/ml streptomycin and 2 mM l-glutamine. The cells were seeded at a density of 1 × 10^5^ cells/cm^2^ on transwell^®^ polycarbonate membranes (12 mm diameter, 0.4 μm pore size) (VWR International BV, Amsterdam, The Netherlands) coated with human placental collagen. The medium (0.5-ml apical compartment, 1.5-ml basolateral compartment) was replaced daily. At day 6 postseeding, the BeWo b30 cell layers were used for transport experiments. The barrier-forming capacity of the BeWo cell layers was evaluated by measuring the transepithelial electrical resistance (TEER) of the cell monolayer using a Millicell ERS-2 Volt-Ohm Meter (Millipore, USA) at day 6 postseeding as described previously (Li et al. 2013). Only the cell layers showing TEER values between 80 and 100 Ω cm^2^ were used for transport experiments.

Transport experiments were initiated by adding 0.5 ml of transport buffer Hank’s balanced salt solution (HBSS, Invitrogen, Breda, The Netherlands) containing the test compound, being 0594, 0595, 0596, 0599, 0600 and 0618, dissolved in DMSO (final solvent concentration 0.5 % DMSO), at concentration of 50 μM, to the apical compartment and 1.5 ml transport buffer to the basolateral compartment. Subsequently, the plate was incubated in a humidified atmosphere with 5 % CO_2_ at 37 °C. After 15, 30, 60 and 90 min, a sample of 0.2 ml was taken from the basolateral compartment and replaced by an equal volume of transport buffer. At the end of each experiment, a 0.2-ml sample was also taken from the apical compartment. Subsequently, the filters with the BeWo b30 cell layers were washed three times with PBS, cut out of the insert, dissolved in 0.25 ml 65 % (v/v) methanol and sonificated for 15 min in a Bandelin Sonorex RK100 (Berlin, Germany) in order to determine the amount of compound accumulated in the cells. After each experiment, mass balance calculations were performed. In each transport study, amoxicillin and antipyrine were included as control and reference compounds. Amoxicillin transport was included to assess for monolayer integrity since it is a compound known to be transported only to a limited extent across the placenta, whereas antipyrine was included as a control for optimal transport and a reference compound to enable calculation of relative transport rates (Li et al. 2013). The transport of the six test compounds, as well as the reference compounds amoxicillin and antipyrine, through the permeable membrane of the transwell filter in the absence of BeWo cells was determined as well. The transport to the basolateral compartment was shown to be equally fast for all compounds (data not shown), ensuring that any differences observed in the transport studies were related to the BeWo cell layer and not to the filter. For each test compound, three independent experiments were performed, with three technical replicates per experiment.

### High-performance liquid chromatography analysis

Samples were analyzed using high-performance liquid chromatography (HPLC) to quantify the amount of test compound in order to determine the transport rate and to perform mass balance calculations. The HPLC system used consisted of a Waters (Milford, MA) 600 controller and a 600 pump, equipped with a photodiode array detector set to record absorption of wavelengths between 200 and 400 nm. A Waters 717 plus autosampler was used for sample injection. The temperature of the autosampler was kept at 7 °C.

For analysis of all compounds, 50 μl sample was injected to a C18 5 μm reverse-phase column (150 mm × 4.6 mm I.D.) with a guard column (7.5 mm × 4.6 mm I.D.) (Alltech, Bergen op Zoom, The Netherlands). The mobile phase used for analysis of all the test compounds consisted of (A) 0.1 % trifluoroacetic acid in nanopure water and (B) HPLC-grade acetonitrile. Elution was at a flow rate of 0.8 ml/min, starting at 22 % B with a linear increase to 100 % B in 8 min. Subsequently, the gradient returned linearly to the initial condition in 10 min and remained 2 min at this condition prior to the next injection. In each analysis, calibration curves of all compounds were included for quantification.

### ES-D3 cell culture

The murine ES-D3 cell line was purchased from ATCC (Wesel, Germany). The cells were maintained in polystyrene cell culture flasks (Corning, The Netherlands) in Dulbecco’s modified Eagle’s medium (DMEM, Invitrogen, Breda, The Netherlands), supplemented with 20 % heat-inactivated fetal calf serum (Lonza, BioWhittaker, Verviers, Belgium), 50 U/ml penicillin with 50 μg/ml streptomycin (Invitrogen), 2 mM l-glutamine (Invitrogen), 0.1 mM *β*-mercaptoethanol (Sigma-Aldrich) and 1 % (v/v) nonessential amino acids (Invitrogen), at 37 °C and 5 % CO_2_ in a humidified atmosphere. Cells were kept undifferentiated with 1000 U/ml murine leukemia inhibiting factor (LIF) (Sigma-Aldrich) and subcultured every 2–3 days using nonenzymatic cell dissociation solution (Sigma-Aldrich) to detach the cells.

### Cytotoxicity assay with ES-D3 cells

To determine cytotoxicity of the compounds, a WST-1 assay was performed. This assay measures the influence of test compounds on the formation of the water-soluble formazan reaction product from WST-1 by mitochondrial succinate-tetrazolium reductase enzymes. The cytotoxicity of a test compound inversely correlates with the absorbance of the produced formazan quantified spectrophotometrically as described previously (Reitsma et al. 2013). ES-D3 cells were exposed to test compounds for the duration of 1 or 5 days as described before (Li et al. 2015). Briefly, cells were seeded in 96-well plates (Greiner bio-one) at concentrations of 20 × 10^4^ cells/ml (1-day exposure) or 1 × 10^4^ cells/ml (5-day exposure) in 100 μl culture medium in the absence of LIF and incubated for 1 day to allow cell adherence. Then, the cells were exposed to the test compounds at concentrations up to 60 μM (final solvent concentration 0.2 % DMSO) and subsequently cultured for 1 or 5 days at 37 °C and 5 % CO_2_ in a humidified atmosphere. Solvent DMSO (0.2 %) was used as a negative control and 1 % Triton X-100 served as a positive control in all cytotoxicity assays. After incubation of 1 or 5 days, 20 μl WST-1 reagent (Roche, Woerden, The Netherlands) was added to each well and the plates were incubated for another 3 h. Then, absorbance was measured at 450 nm using a SpectraMax M2 (Molecular Devices, Sunnyvale, USA). Three wells were used per treatment in each independent experiment. Three independent experiments were done for each compound. The cell viability was expressed as % of the solvent control, with the solvent control set at 100 % viability. Reproducible results were obtained from the treatments of negative and positive controls in all of the cytotoxicity assays.

### Differentiation assay with ES-D3 cells

Differentiation assays were carried out to detect the effect of test compounds on the differentiation of ES-D3 cells into contracting cardiomyocytes using culture medium in the absence of LIF. On day 1, droplets of 20 μl cell suspension (3.75 × 10^4^ cells/ml) were placed as hanging drops, to which the test compounds 0594, 0595, 0596, 0599, 0600 and 0618 were added at concentrations ranging from 0.2 to 60 μM (final solvent concentration 0.2 % DMSO), on the inner side of the lid of a 96-well plate. Sterile lids of Eppendorf tubes were placed on the corner wells of the plates to prevent contact of the drops with the plate. The wells of the 96-well plate were filled with 250 μl phosphate buffered saline (PBS) (Invitrogen), and the plate was sealed with Micropore tape (3M, Neuss, Germany) to prevent evaporation of the hanging drops. Plates were incubated for 3 days at 37 °C and 5 % CO_2_ in a humidified atmosphere. In the drops, cells formed embryonic bodies (EBs), which were transferred to nontissue culture-treated Petri dishes (diameter 6 cm, Greiner) with 5 ml of medium with test compound. On day 5, the EBs were transferred to a 24-well plate (Corning) with 1 ml of medium with test compound, with one EB per well. On day 10, the number of wells containing contracting EBs was determined by visual inspection using a light microscope. Solvent control (0.2 % DMSO in culture medium) was included in each experiment. The solvent control was also used to assess the quality of the batch of ES-D3 cells used in each individual test (cells being randomly distributed over test groups and solvent control). Tests were accepted for further analysis if at least 21 of the 24 wells of the solvent control contained contracting cardiomyocytes. For each test compound, three independent assays were performed. The results were expressed as “fraction of total,” with 1.0 implying all EBs in one 24-well plate differentiated into contracting cardiomyocytes.

### In vivo experiments

The in vivo investigations of maternal and prenatal developmental toxicity were performed as a screening approach following the general principles of OECD 414 and OPPTS 870.3700 test guidelines, and the OECD and US Environmental Protection Agency Good Laboratory Practice Standards [40 CFR Part 160 (FIFRA) and Part 792 (TSCA)]. The major differences to a full guideline study were the smaller sample size, using 9–10 instead of at least 16 pregnant rats, per test substance. Furthermore, the fetal evaluation was performed using 4–10 l, the lower number being used if sufficient (i.e., positive) results were obtained to assess the prenatal developmental toxicological potential of the test compounds. The in vivo studies were performed according to the German Animal Welfare Act, the European Council Directive 2010/63/EU and in an AAALAC-accredited facility. This screening study of developmental compounds was approved by the local authorizing agency for animal experiments (Landesuntersuchungsamt Koblenz, Germany) as referenced by the approval number 23 177-07/G08-3-008).

### Test animals

The animals were paired by the breeder [time-mated Wistar rats, Crl: WI (Han), Charles River Laboratories, Sulzfeld, Germany], between 10 and 12 weeks of age, and supplied on the day of evidence of mating; this day is referred to as gestational day (GD) 0 and following day as GD 1. All animals showed no clinical signs of disease. This strain has been extensively used, both in our laboratory and elsewhere, and is sensitive to reproductive toxicants. All rats were housed individually in Makrolon cages with Lignocel PS 14 fibers dust-free bedding and wooden enrichment blocks. The cages were kept in climate-controlled rooms at 20–24 °C with a relative humidity of 30–70 %, an air exchange rate of 15 times per hour and a 12-h light/dark cycle. Diet (Ground Kliba SA, Switzerland) and tap water were provided ad libitum. In-life data (mortality, clinical signs, body weights and food consumption) were recorded throughout the study, but presented only if relevant for the interpretation of prenatal developmental effects.

### Experimental procedure

Studies were conducted with daily oral administration of test substances by gavage (test compounds 0594, 0595, 0596, 0599 and 0600) or via diet (test compound 0618) from GD 6 to GD 19 (Table 4). The individual dose levels were selected based on the results of a repeated exposure to nonpregnant female rats over 14 days (data not shown). According to the OECD 414 test guideline, it was aimed to cause signs of maternal toxicity as recommended at the high-dose level. The test compounds administered by gavage were administrated between 100 and 600 mg/kg bw/day using the vehicle 1 % carboxymethylcellulose in deionized water. The standard dose volume was 10 ml/kg bw. The test compound 0618 was administered via the diet at dose levels of 300 ppm (dose was reduced from 2500 ppm on GD 6 and 7 causing severe clinical signs) and 1000 ppm in the diet. The exposures were corresponding to a substance intake of 28 mg/kg bw/day (GD 8–19) and 69 mg/kg bw/day (GD 6–19), respectively.

### Necropsy and fetus preparation

On GD 20, the surviving dams were anesthetized with isoflurane, killed by decapitation and examined macroscopically. For each dam, the uterus was opened and the number, distribution and classification of implantation sites (live fetus, early and late fetal resorptions, and dead fetus) were determined. The fetuses were removed, sexed and fetal body weight determined. Gross pathological examination of the fetuses, including assessment of abnormalities of the fetal membranes, placentas, amniotic fluid and umbilical cord, was performed. Subsequently, all fetuses were killed by injection of pentobarbital. About half of the fetuses of each dam were fixed in ethyl alcohol and after fixation, stained according to a modified method of Dawson (1926) to show the skeleton. The other half of the fetuses of each dam was fixed in Harrison’s fluid. After fixation, the soft tissue of these fetuses was examined according to a modified microdissection method (Barrow and Taylor 1969). The glossary of Wise et al. (1997) and its updated version of Makris et al. (2009) were essentially used to describe findings in fetal morphology. Classification of these findings was based on the terms and definitions proposed by Chahoud et al. (1999) and Solecki et al. (2001, 2003). A permanent structural change that is likely to affect adversely the survival or health was assessed as malformation. A change that also occurs in the fetuses of control animals and/or is unlikely to affect adversely the survival or health was assessed as variation. This includes delays in growth or morphogenesis that have otherwise followed a normal pattern of development.

### Data analysis

#### BeWo transport data

For each compound, the linear appearance rate in the basolateral compartment was determined. These linear appearance rates were used to calculate apparent permeability (Papp) coefficients [Papp coefficient (cm/s) = (Δ*Q*/Δ*t*)/(*A* × *C*_0_)], where Δ*Q* is the amount of test compound (nmol) transported to the receiver chamber in a certain time span [Δ*t* (s)], *A* is the cell surface area (cm^2^) and *C*_0_ is the initial concentration of the test compound (µM). To calculate Δ*Q*, a correction was made to compensate for the removal of compound when taking samples [Δ*Q* at *t*_x+1_ = amount measured at t_x+1_ (nmol) (basolateral concentration at *t*_x+1_ (μM) × 1.5 (ml)] supplemented with the amount removed at *t*_x_ (nmol) [basolateral concentration sample at *t*_x_ (μM) × 0.2 (ml)]. Subsequently, relative Papp values were determined by expressing the Papp coefficient as a fraction of the Papp coefficient obtained for antipyrine.

#### In vitro ES-D3 differentiation data

Different dichotomous concentration–response models were fitted to the in vitro developmental toxicity data obtained from the ES-D3 cell differentiation assay to calculate benchmark concentrations (BMC) using Environmental Protection Agency benchmark dose (BMD) software version 2.4. For each test compound, the BMC_d_50, representing the concentration for a 50 % reduction in the number of differentiated EBs, was derived. Models included in the evaluation were the gamma, logistic, loglogistic, probit, logprobit, multistage, weibull and the quantal-linear model. Goodness of fit of the models was evaluated to accept a model, based on the *p* values, the scaled residuals and the graphical displays obtained. The lowest BMC_d_50 value was chosen from the accepted models.

Figures of concentration–response curves for both differentiation and cytotoxicity were made using GraphPad Prism 5 using a four-parameter logistic model. These curves were not used for the derivation of the BMD_d_50 values since BMD_d_50 values were derived as described above using BMD modeling.

To combine in vitro developmental toxicity data obtained from the ES-D3 cell differentiation assay with placental transfer data obtained from the BeWo transport model, a corrected BMC_d_50 value was calculated by dividing the BMC_d_50 values by the relative Papp values, as described in Li et al. (2015).

#### In vivo data

Data obtained for food consumption, body weight, carcass weight, weight of unopened uterus, weight of placentas and fetuses, the number of implantations, number of late fetal resorptions and percentage of postimplantation loss were analyzed by a simultaneous comparison of all dose groups with the control group using Dunnett’s test (Dudewicz et al. 1975; Dunnett 1964). The number of pregnant animals at the end of the study, mortality rate of the dams and number of litters with fetal findings were analyzed by Fisher’s exact test (Siegel 1956) and the proportion of fetuses with findings per litter by Wilcoxon signed-rank test (Hettmansperger and McKean 1978; Siegel 1956). Maternal toxicity was classified as slight if body weight and/or carcass weight reduction is not above 10 %, moderate if body weight and/or carcass weight reduction is between 10 and 20 % and severe if body weight and/or carcass weight reduction is above 20 %. All the in vivo data are expressed as the affected fetuses/litter.

## Results

### In vitro BeWo transport

**Fig. 1:**
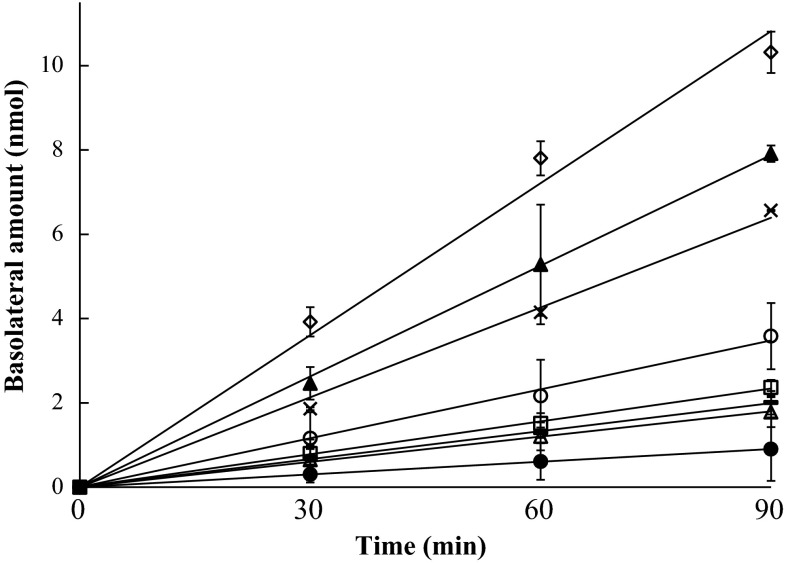
Amount of 0594 (*open circle*), 0595 (*filled circle*), 0596 (*filled triangle*), 0599 (*times symbol*), 0600 (*filled square*), 0618 (*hyphen symbol*), amoxicillin (*open triangle*) and antipyrine (*open diamond symbol*) in the basolateral compartment in the in vitro BeWo model with increasing time using initial concentrations of 50 μM (25 nmol) in the apical compartment. Data are presented as mean ± SD (*n* = 3)

For all transport experiments, the mass balances were between 91 and 99 %. Figure 1 shows the increasing amount of test compounds in the basolateral compartment of the BeWo model over time, after adding 25 nmol to the apical compartment. Antipyrine was included as a reference compound known to be efficiently transported across the BeWo cell layer, and amoxicillin was used as a control compound to check the integrity of the cell layer (Li et al. 2013). The slow transfer of amoxicillin indicated an intact BeWo cell layer. For up to 60 min, the transport of all compounds to the basolateral compartment was linear in time. Therefore, the linear appearance rate of compound in the basolateral compartment could be determined using data at 30 min for the calculation of Papp coefficients. The data in Table 2 show a wide (up to eightfold difference) range of Papp coefficients for the six test compounds, illustrating different placental transfer rates among them, with 0596 being transported at the highest rate and 0595 at the lowest rate. Besides the transport rate, intracellular accumulation of six test compounds and the control compounds at 90 min was quantified and the results obtained are shown in Table 2. The data show that all triazoles tended to accumulate in the BeWo cells and that amoxicillin and antipyrine did not. Higher amounts of 0595, 0600 and 0618 were detected in the cells than of the other three triazoles.

**Table 2:** Papp coefficients at 30 min and relative Papp values of six test compounds and the reference compounds amoxicillin and antipyrine in the BeWo model

Compounds	Intracellular accumulation (% of added amount)	Papp coefficient (10^−6^ cm/s)	Relative Papp value
Amoxicillin	0	6.5 ± 0.2^a^	0.16
0594	24	11.5 ± 2.6	0.30
0595	79	3.0 ± 0.4	0.08
0596	12	24.4 ± 2.7	0.63
0599	22	18.4 ± 1.1	0.47
0600	66	7.9 ± 1.0	0.20
0618	56	7.1 ± 0.2	0.18
Antipyrine	0	38.9 ± 3.0	1.00

^a^Mean ± SD

### Cytotoxicity assay with ES-D3 cells

**Fig. 2:**
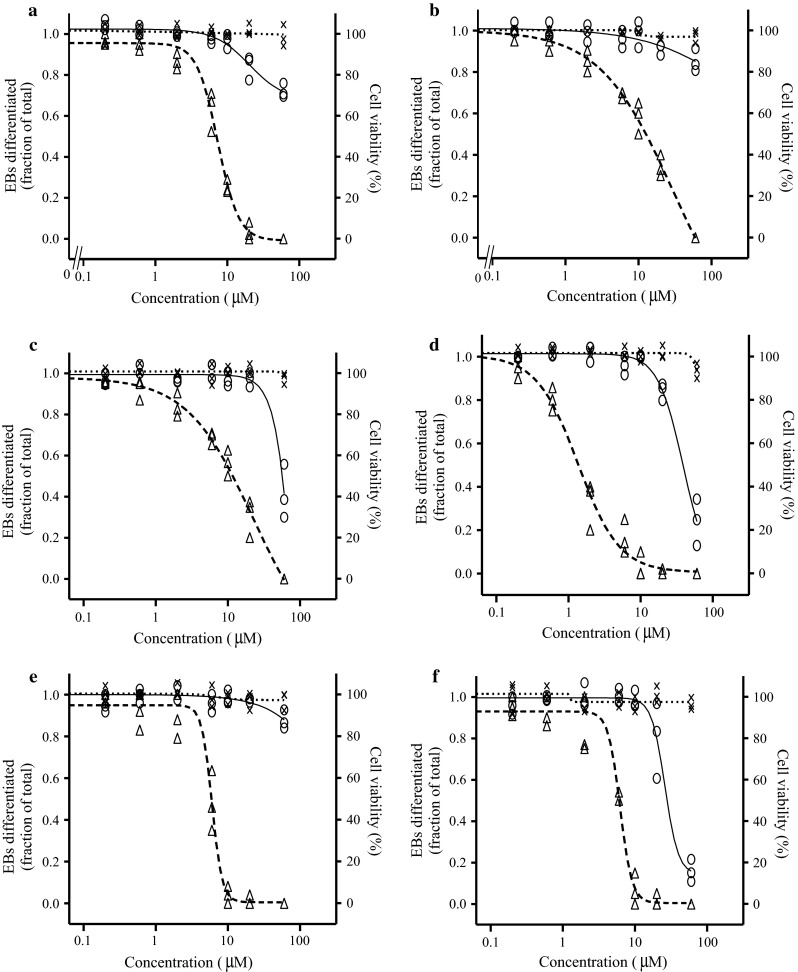
Concentration-dependent effects of test compounds 0594 (**a**), 0595 (**b**), 0596 (**c**), 0599 (**d**), 0600 (**e**) and 0618 (**f**) on cell viability for 1-day (*times symbol*) and 5-day (*open circle*) exposure and on inhibition of ES-D3 cell differentiation (*open triangle*). Figures present data of three independent experiments

WST-1 assays for both 1- and 5-day exposure were performed to evaluate the cytotoxic effects of the compounds on the ES-D3 cells (Fig. 2). For all compounds, the concentrations tested (up to 60 μM) were noncytotoxic as determined in the 1-day cytotoxicity assay. Among the six compounds, 0618 was the most potent one in the 5-day cytotoxicity assay, reducing the cell viability to 10 % at 60 μM. Exposure of 60 μM 0599, 0596 and 0594 to the ES-D3 cells resulted in 80, 70 and 30 % decline in cell viability, respectively, reflecting lower cytotoxic properties. The least cytotoxic triazoles were 0595 and 0600, which caused slight reduction in cell viability (20 %) up to 60 μM.

### Differentiation assay with ES-D3 cells

To study the in vitro developmental toxicity of the antifungal triazoles, the effect of the compounds on the differentiation of ES-D3 cells into contracting cardiomyocytes was evaluated. All test compounds induced a concentration-dependent inhibition of the differentiation of the ES-D3 cells into contracting cardiomyocytes (Fig. 2). The calculated BMC_d_50 values were at concentrations that did not cause cytotoxicity (after 1 and 5 days), indicating that inhibitory effects on the differentiation of EBs are not due to cytotoxicity of the test compounds. BMC_d_50 values are summarized in Table 3, showing that 0599 was the most potent in inhibiting the differentiation of EBs, followed by 0600 > 0618 > 0594 > 0595 > 0596.

**Table 3:** BMC_d_50 values for in vitro developmental toxicity of test compounds in the ES-D3 cell differentiation assay and corrected BMC_d_50 values obtained by combining the ES-D3 cell differentiation assay data with data on placental transfer from the BeWo transport model

Novel triazoles	BMC_d_50 (µM)	Corrected BMC_d_50^a^ (µM)
0594	6.9	23.3
0595	11.0	141.8
0596	11.4	18.1
0599	1.8	3.8
0600	4.2	21.0
0618	4.3	23.7

^a^Corrected BMC_d_50 value was calculated by dividing the BMC_d_50 values by the relative Papp values

### Combination of in vitro developmental toxicity data with BeWo transport data

To combine in vitro developmental toxicity data obtained from the ES-D3 cell differentiation assay with placental transfer data obtained from the BeWo transport model, a corrected BMC_d_50 value was calculated by dividing the BMC_d_50 values by the relative Papp values (Table 3). After the correction, the potency ranking was altered, being 0599 > 0596 > 0600 > 0594 > 0618 > 0595.

### Potency ranking derivation from in vivo data

In the in vivo studies, all tested compounds showed, to some degree, a potential to cause prenatal developmental toxicity. All statistically significant and toxicologically relevant alterations relative to controls are summarized in Table 4. The potency of each of the test compounds to cause prenatal developmental toxicity was assessed based on the observed fetal findings, including teratogenic effects, taking into account the presence or absence of maternal toxicity at the different dose levels.

**Table 4:** Developmental data on the incidence of cleft palate or skeletal malformation in rat

Compound	Exposure route and duration	Dose	Maternal toxicity			Placenta weight^b^ (%)	Early resorption^b^ (%)	Late resorption^b^ (%)	Postimplantation loss^b^ (%)	Developmental toxicity					Ranking
			No. of females (mated/pregnant)	Findings^b^	Classification					Total number of fetuses	Fetal weight^b^ (%)	No. of fetuses/litters used in skeletal examination	Malformation^c^	Three most observed malformation^c^	
0594	Gavage GD 6–19	600 mg/kg	10/10	FC (6–8) 86 %*	Slight	116	14.1	0.8	14.9*	98	101	19/4	Total 12.5 %	Bent femur 12.5 %	4
		200 mg/kg	10/10	No	No	110	5.9	1.9	7.8	99	107	–	–	–	
0595	Gavage GD 6–19	600 mg/kg	10/9	Mortality 30 % BW 74 %** CW 89 %*	Severe	–	100.0**	0.0	100.0**	0	–	–	–	–	6
		200 mg/kg	10/9	NAD	No	117	21.9*	2.0	24.0*	74	102	18/4	Total 0 %	–	
0596	Gavage GD 6–19	600 mg/kg	10/10	NAD	No	133**	7.7	0.8	8.6	102	105	57/10	Total 100 %**	Severely malformed skull bones 82.8 %** Misshapen cervical arch 70.2 %** Misshapen basisphenoid 13 %	2
		200 mg/kg	10/10	NAD	No	127**	2.9	0.0	2.9	109	108	54/10	Total 47 %**	Misshapen basisphenoid 30.6 %** Misshapen cervical arch 14.3 %* Severely malformed skull bones 5.3 %	
0599	Gavage GD 6–19	250 mg/kg	10/10	Mortality 100 %**	Severe	110			–	0	–		–	–	1
		100 mg/kg	10/10	FC(6–20) >74 %** BWC (6–8) 66 %** CW 91 %*	Slight	116	7.8	0.8	8.6	99	102	21/4	CP 10 % Total 100 %**	Misshapen temporal bone 85.4 %** Misshapen basisphenoid 74.2 %** Small tuberositas deltoidea 66.3 %**	
0600	Gavage GD 6–19	600 mg/kg	10/10	BWC (6–8) 27 %**	Slight	130**	6.0	1.0	7.0	104	102	22/4	CP 5 % Total 81 %**	Small tuberositas deltoidea 48.1 %** Absent tuberositas deltoidea 44.4 %** Misshapen pterygoid bones 36.3 %*	3
		200 mg/kg	10/10	NAD	No	105	4.0	0.0	4.0	99	102	20/4	Total 0 %	–	
0618	Diet GD 6–20	1000 ppm	10/10	BW 87 %** CW 83 %**	Moderate	162**	7.2	23.2**	30.3**	74	87**	57/10	Total 2.5 %	Bent femur 2.5 %	5
		300/(2500) ppm^a^	10/10	CW 95 %	Slight	111	4.6	0.8	5.4	110	101	41/10	Total 1.4 %	Misshapen basisphenoid 1.4 %	

*GD* gestation day, *FC* food consumption, *BW* body weight, *CW* carcass weight, *BWC* body weight change, *CP* cleft palate, *NAD* nothing abnormal detected

– Data not available

* *p* ≤0.05

** *p* ≤0.01

^a^2500 ppm reduced to 300 ppm on GD 8 of first cohort (two animals) and GD 7 of second cohort (eight animals)

^b^Statistics: Dunnett test (two-sided)

^c^Statistics: Wilcoxon test (one-sided)

The highest potential to cause prenatal developmental toxicity, based on the results of the in vivo studies, was observed for the test compound 0599 (ranking 1). It caused teratogenic effects in 100 % of fetuses, manifested in skeletal malformations on the tuberositas deltoidea and pterygoid bones as well as palate at 100 mg/kg bw/day. This dose level did not alter the overall development of the fetuses indicated by their body weight or in utero survival indicated by resorption rate. At this exposure, maternal toxicity was observed noted as decreased food consumption (74 % GD 6–20), body weight change (66 % GD 6–8) and carcass weight (91 %). Maternal toxicity is not considered to have contributed significantly to the fetal effects.

The second highest potential to cause prenatal developmental toxicity was observed for the test compound 0596 (ranking 2). This compound also caused teratogenic effects in all fetuses, manifested in skeletal malformations, like the test compound 0599. The malformation observed at the highest incidences was observed in skull bones (including basisphenoid) and cervical arches. But this time all fetuses were only affected at a six-time higher dose level of 600 mg/kg bw/day. Comparable to the test substance 0599, this dose level did not affect the overall development of the fetuses indicated by their weight or in utero survival indicated by resorption rate being comparable to the control. The teratogenic effects were observed at a dose level causing almost no maternal toxicity, only visible in an increased placental weight (133 %). At 200 mg/kg bw/day, the pattern of treatment-related effects was comparable to 600 mg/kg bw/day, but malformations being observed in 47 % of fetuses per litter.

The third highest potential to cause prenatal developmental toxicity was observed for the test compound 0600 (ranking 3). It was causing treatment-related findings neither on fetuses nor on dams at 200 mg/kg bw/day. At 600 mg/kg bw/day, the teratogenic potential of the test compound was manifested in an increased incidence of skeletal malformations (81 % of fetuses per litter) without affecting the fetal growth or causing increased number of resorptions. The significantly increased malformation rate was observed in small or absent tuberositas deltoidea, misshapen pterygoid bones and cleft palate. The maternal toxicity observed in lower body weight changes in the beginning of exposure (27 % GD 6–8), and the increased placental weight (130 %) might had enhanced the severity of teratogenic effects but could not be encountered for their occurrence only.

The third lowest potential to cause prenatal developmental toxicity was observed for the test compound 0594 (ranking 4). Like the test compound 0600, it did not cause treatment-related findings in fetuses and dams at 200 mg/kg bw/day. At 600 mg/kg bw/day, only slight maternal toxicity was observed in minor alterations of food consumptions (86 % GD 6–8) without any effect on the body weight development of the dams. A borderline increase in postimplantation loss was determined with 14.9 % being only slightly above the spontaneous incidence of 14.7 % given in the historical control data for this rat strain in our test facility. In comparison with test compound 0600, a weaker teratogenic potential of the test compound 0594 was observed manifested in an increased total skeletal malformation incidence of 12.5 % fetuses per litter (bent femurs) at 600 mg/kg bw/day. This dose did not significantly affect number of fetuses alive per litter or the growth of the fetuses.

The second lowest potential to cause prenatal developmental toxicity was observed for the test compound 0618 (ranking 5). It caused slight or moderate maternal toxicity at 300 and 1000 ppm manifested in significantly decreased body (87 % at 1000 ppm) and/or decreased carcass weight (95 % at 300 ppm and 83 % at 1000 ppm). The highest dose level tested also caused significantly increased placental weights (162 %) and postimplantation loss (30.3 %). The latter was based mainly on the increased number of late resorptions (23.2 %). The observed decreased fetal development manifested in the lower fetal body weight (87 % at 1000 ppm) could partially be explained by the moderate maternal toxicity observed. However, the skeletal malformation observed here, low incidences of bent femur, is a rare finding and is not in the historical control data. Thereby, the test compound 0618 still demonstrated a weak teratogenic potential.

The lowest potential to cause prenatal developmental toxicity was observed for the test compound 0595 (ranking 6). It has a relatively high general toxic potential leading to poor general state in 3 out of 10 rats after 10–11 days of exposure to 600 mg/kg bw/day. These animals had been killed moribund on GD 15 and 16. The surviving dams showed severe maternal toxicity in decreased body (74 %) and carcass (89 %) weight. At this dose level, none of the embryos survived the in utero exposure leading to 100 % early resorptions. At 200 mg/kg bw/day, no maternal toxicity but still a significant increase in postimplantation loss (24 %) was observed mainly caused by an increase in number of early resorptions (21.9 %). The surviving fetuses did not show alterations on growth or malformations. Thereby, no teratogenic potential was observed for the test substance 0595 in this in vivo study.

Based on this assessment, a developmental potency ranking of the test compounds was obtained: 0599 > 0596 > 0600 > 0594 > 0618 > 0595.

### Comparison of in vivo and in vitro developmental toxicity ranking

Both in vitro developmental toxicity potency rankings based on BMC_d_50 alone and the placental transfer corrected BMC_d_50 values were compared with in vivo potency ranking, to assess the usefulness of combining data from the ES-D3 cell differentiation assay with data from the BeWo transport model to predict in vivo potency ranking (Table 5). Based on the in vivo ranking, 0599 was the most potent compound in vivo and 0595 was the least potent one. The potency ranking of the six test compounds in the ES-D3 cell differentiation assay correlates to some extent with the in vivo ranking, with 0599 being the most potent one, however, with clear differences for the less toxic ones. For 0596, especially, the relative potency is not well predicted in the ES-D3 assay, being least toxic in the ES-D3 cell differentiation assay, whereas almost most toxic in vivo. When the ES-D3 cell differentiation data are combined with the results obtained in the BeWo transport model to obtain corrected BMC_d_50 values, a better correlation with the in vivo ranking was obtained with no discrepancy (Table 5). These data thus demonstrate the power of including a component of kinetics when predicting relative in vivo toxicity potencies based on in vitro toxicity data.

**Table 5:** Comparison of the in vivo developmental toxicity ranking of test compounds with the ES-D3 cell differentiation alone or with the ES-D3 cell differentiation assay combined with the BeWo transport model

Methods	Toxicity ranking					
	Least toxic		→			Most toxic
ES-D3	0596	0595	0594	0618	0600	0599
ES-D3 + BeWo	0595	0618	0594	0600	0596	0599
In vivo	0595	0618	0594	0600	0596	0599

## Discussion

The ES-D3 cell differentiation assay has shown to be a promising method to assess the developmental toxicity potency ranking of series of structurally related compounds in vitro, and it has been suggested that the capacity of this assay to predict in vivo potency ranking could be improved by combining kinetic information with the in vitro data (De Jong et al. 2009; Louisse et al. 2011; Strikwold et al. 2012). We have previously demonstrated that the ES-D3 cell differentiation assay combined with the in vitro BeWo transport model for placental transfer is able to better predict the in vivo developmental toxicity of a set of reference azoles, than as a stand-alone assay (Li et al. 2015). In this study, we extended the dataset, to validate this combined model by testing more compounds within the series of antifungal triazoles.

The in vitro BeWo transport model was used to obtain the relative transport rate in the present study, as it was previously shown to be suitable to adequately characterize relative placental transfer rates of compounds (Carreira et al. 2011; Li et al. 2013; Poulsen et al. 2009). The six test compounds showed different transfer rates (up to eightfold) through the placental barrier in the BeWo model, indicating that transport velocities to the fetus in vivo may differ as well. However, how this exactly translates to differences in fetal exposure is not known, due to the fact that in vivo experimentation of placental transport in humans is not feasible on a large scale for obvious ethical reasons and also because little has been published on the fetal bioavailability of antifungal compounds in vivo. We hypothesize that the extent of fetal exposure would be positively correlated with the relative transport velocity in the BeWo model. Therefore, we investigated whether the correlation between in vitro BMC_d_50 value-based potency ranking and in vivo developmental toxicity potency ranking would improve when we corrected the in vitro BMC_d_50 values for differences in placental transfer, by dividing these BMC_d_50 values for the six test compounds in the ES-D3 cell differentiation assay by relative Papp values obtained in the BeWo model.

With the ES-D3 cell differentiation assay alone, the predicted toxic concentrations among the six test compounds were within the same order of magnitude, but the combined assays resulted in a different ranking, with the most potent compound being about 30 times more toxic than the least potent one. The results obtained reveal that the ranking according to the corrected BMC_d_50 values correlated better than the uncorrected ones with the ranking that could be derived from in vivo developmental toxicity data. This shows that combining the ES-D3 cell differentiation assay results with placental transfer kinetic data improved the ability of this in vitro assay to predict in vivo developmental toxicity potencies of the tested compounds. It should be noted that other important in vivo kinetic processes than placental transfer, such as intestinal absorption, maternal metabolism and placental metabolism, were not taken into account and that these will contribute to the amount of compound available in the fetus. Therefore, incorporating more kinetic information is likely to further improve the predictive value of the ES-D3 cell differentiation assay. Besides, the BeWo cell model may not be suitable to determine the absolute transport rates of compounds, given that the BeWo cell model is a simplification of the in vivo placental transfer system. In those cases where it is important to use exact fetal bioavailability values, physiologically based kinetic (PBK) models, which describe the in vivo absorption, distribution, metabolism and excretion processes of a compound should be used for the translation of in vitro toxicity data to the in vivo situation (Louisse et al. 2010, 2014; Strikwold et al. 2013).

We acknowledge that the increase in placental weight, noted for 0596 (at 200 and 600 mg/kg bw/day), 0599 (at 600 mg/kg bw/day) and 0618 (at 1000 ppm) in the rat studies cannot be reflected in the ES-D3 assay, and therefore, these effects were not taken into account when making the comparison among the different triazoles. The increased placental weight in pregnant rat may be the result of inhibition of aromatase enzyme activities (Stinchcombe et al. 2013). The contribution of placental changes induced by azole compounds to fetal development and its relevance to humans is currently under investigation (Rey Moreno et al. 2013).

In this study, intracellular accumulation of test compounds in the placental cells was investigated in the BeWo cell model. Triazoles 0595, 0600 and 0618 showed a high percentage of accumulation, up to 79 % of the total mass added, in the BeWo cells. If this accumulation in placental cells is also occurring in vivo, the accumulated compounds may potentially affect the development or function of the placenta, given that the placenta is an entry organ to the fetus and vulnerable to the adverse effects of many toxicants. Structural or functional damage to the placenta can lead to adverse effects, such as abortion, birth defects and stillbirth. We observed that triazole 0595, which showed the highest accumulation in the BeWo cells, induced the highest level of postimplantation loss, making it tempting to speculate that there might be a link between the BeWo cellular accumulation and the in vivo resorption rate. However, as BeWo cells only represent part of the placental tissue and since placental tissue changes over pregnancy, it is difficult to translate this in vitro finding to the exact in vivo situation. Results on placental accumulation of the six triazoles in vivo are not available, but it has been elucidated that some pesticides were found to accumulate in placental tissue (Mohan and Singh 2013; Saxena et al. 1981). Therefore, it might be of interest to pay attention to possible placental toxicity, for which the in vitro BeWo cell model might be useful for prioritization because as shown in the present study the model is able to provide information on the intracellular accumulation of compounds.

The ES-D3 cell differentiation assay is considered to represent the fetal component of developmental toxicity. However, the (azole) antifungal compounds are found to have the ability to inhibit cytochrome enzymes (Marotta and Tiboni 2010) and in particular may inhibit estrogen biosynthesis through CYP19 aromatase inhibition, reducing the conversion of androgens to estrogens and exerting endocrine disrupting effects (Kjærstad et al. 2010b). This process takes place in the maternal organism, and the phenotypic analysis of the inhibition of cardiac differentiation in the ES-D3 cell differentiation assay cannot take this part of developmental toxicity into account. Therefore, other relevant in vitro assays, such as the steroidogenesis assay (Kjærstad et al. 2010b; Stresser et al. 2000), may be needed. In addition, although we have proven in the previous and the present study that the ES-D3 cell differentiation assay combined with the BeWo transport assay shows to be an appropriate in vitro toxicological system to achieve an enhanced predictivity of the relative developmental toxicity for antifungal compounds, one should still note that one single alternative testing approach is not likely able to predict for the entire scope of developmental toxicants. To obtain information on mechanisms underlying developmental toxicity, the ES-D3 cell differentiation assay could be combined with transcriptomics analyses, which can be used to group compounds in classes based on toxicity mechanisms (van Dartel and Piersma 2011). A tool box which integrates different relevant in vitro assays into an integrated testing strategy may provide the best possible strategy to characterize both the toxicological profile and the relevant mechanistic information for human chemical risk assessment.

When it comes to risk assessment, it should be remembered that many antifungal products contain mixtures of active substances, for example, for broad-spectrum disease control or for resistance management to obtain the broadest benefits possible (Kjærstad et al. 2010a). Though current risk assessment approaches are predominantly based on individual azole fungicides, human exposure is in general to complex mixtures of pesticides. It is therefore of interest to investigate the developmental potency of the mixture of antifungal compounds in the future research.

In conclusion, in the present study we provided further evidence that the ES-D3 cell differentiation assay, combined with the in vitro BeWo transport model, is able to better predict the in vivo developmental toxicity ranking of the antifungal compounds, than as a stand-alone assay. At this stage, the applicability domain of this combined in vitro toxicodynamics and toxicokinetics approach is not known. Other series of compounds from different chemical classes should be tested to further evaluate the capacity of this approach in predicting developmental toxicity. Since the method uses only in vitro assays to predict in vivo developmental toxicity ranking of chemicals, it can contribute to the 3Rs (replacement, reduction and refinement) of animal testing. The predictive ability of the ES-D3 cell differentiation assay can most likely be further enhanced if it is combined with more kinetic data and PBK modeling.
